# Accuracy, Consistency, and Reproducibility of the Triaxial Accelerometer in the iPod Touch: A Pilot Study

**DOI:** 10.2196/mhealth.3008

**Published:** 2014-11-24

**Authors:** Christopher Khoo Chee Han, Rukmanikanthan AL Shanmugam, David Choon Siew Kit

**Affiliations:** ^1^Department of Orthopaedic SurgeryFaculty of MedicineUniversity MalayaKuala LumpurMalaysia

**Keywords:** accelerometry, tri-axial accelerometer, iPod Touch

## Abstract

**Background:**

The use of a mobile consumer communicative device as a motion analysis tool for patients has been researched and documented previously, examining the triaxial accelerometer embedded in such devices. However, there have been few reports in the literature testing the sensitivity of an embedded triaxial accelerometer.

**Objective:**

Our goal in this study was to test the accuracy, consistency, and reproducibility of the triaxial accelerometer in the iPod Touch.

**Methods:**

In this pilot study, we subjected the triaxial accelerometer in the iPod Touch to a free fall from a height of 100 cm in order to test its accuracy, consistency, and reproducibility under dynamic conditions.

**Results:**

The resultant vectorial sum acceleration was mean 0.999 g (standard gravity; SD 1.51%; 95% CI 0.99-1.01), indicating very high accuracy and sensitivity under dynamic conditions.

**Conclusions:**

Our results highlighted the reproducibility of the capability of the triaxial accelerometer in the iPod Touch to capture data accurately and consistently. Thus, the device has huge potential as a motion analysis tool for measuring gait and studying balance and mobility in patients before and after surgery.

## Introduction

The world is witnessing a huge increase in the mobile electronic devices that are used in everyday activities. The 4th generation iPod Touch manufactured by Apple Inc. is one such device that has had a tremendous impact on our lives, serving as a communication device, as well as a mobile personal assistant. This device is equipped with a built-in triaxial gyroscope and accelerometer, which have been shown by multiple researchers to be capable of detecting, with good accuracy, daily activities such as walking, jogging, sitting, and even playing cricket [1-4]. Recent literature also supports the reliability of various smartphone devices in the measurement of gait parameters and as an analytical tool to study balance and mobility [5-7].

The built-in triaxial accelerometer in an iPod comes in the form of a microelectromechanical system (MEMS). MEMS is the technology of very small devices. MEMS are made up of subcomponents between 1 to 100 micrometers in size (ie, 0.001 to 0.1 mm), and generally range in size from 20 micrometres (20 millionths of a meter) to 1 millimeter (ie, 0.02 to 1.0 mm). They consist of a central unit that processes data (the microprocessor) and several components that interact with the surroundings such as microsensors like accelerometers and gyroscopes. MEMS accelerometers allow measurement of the instantaneous acceleration of an object, compared to gravity at any given time, in a free-fall reference frame both in the static and dynamic motion characteristics. They generally allow measurement of the acceleration in 3 axes, hence the name triaxial accelerometer.

However, the sensitivity of the MEMS accelerometer intalled in the iPod has not been determined with a sufficient level of accuracy, consistency, and reproducibility in the literature. Recently, a small-scale pilot study demonstrated consistent sensitivity values in measuring instantaneous accelerations in a steady state across multiple iPods [8]. The testing for sensitivity, accuracy, and reproducibility of the MEMS accelerometer in the iPod under a dynamic state had not been performed before. We therefore embarked on this study to determine the accuracy, consistency, and reproducibility of the built-in MEMS triaxial accelerometer housed within an iPod Touch by determining its vectorial sum acceleration when subjected to a free fall drop from a height of 100 cm and to compare the results with the established standard of 1 g (standard gravity, equivalent to 9.81 m/s^2^) under these circumstances.

## Methods

### Overview

**Figure 1:**
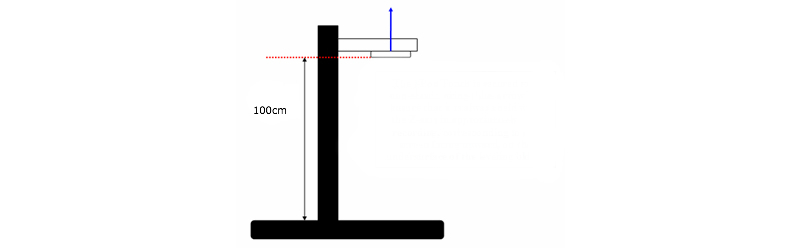
This is a diagram of the set-up for the iPod Touch free fall test showing the distance of travel of 100 cm. The i-Pod Touch is secured to a non-elastic string (blue arrow) to ensure that it is always held with the Z-axis in approximately -1G recording, corresponding to the screen facing upward, on the undersurface of the levelling block.

A single 4th generation iPod Touch mobile device was used to collect instantaneous acceleration in a free fall state from a height of 100 cm. The device was secured to a protective non-elastic string and placed on the under surface of a leveling block measured 100 cm from the ground with the z-axis in approximately -1 g recording position, which corresponds to the screen facing upward towards the ceiling (Figure 1).

Prior to the start of the experiment, we calibrated the triaxial accelerometer software (Accel4Pros) pre-installed in the iPod. The Accel4Pros triaxial accelerometer software captures acceleration range within -2.3 g to +2.3 g with a data capture frequency of 20 data per second or 20 hertz. We then dropped the iPod towards the ground while the program was running; 30 trials of free fall were performed. Following each trial, the acceleration data were stored on the device in Comma Separated Value format and exported via Internet to a remote email account where it was downloaded and interpreted using Microsoft Excel.

### Statistical Analysis

Triaxial accelerometer data analysis was carried out prior to performing the statistical analysis. The mathematical formula for calculation of vectorial sum acceleration, r, using acceleration data from the triaxial accelerometer, is the root square of the sum of (X^2^ + Y^2^ + Z^2^), as shown below:

r=√ (X^2^ + Y^2^ + Z^2^)

where r=vectorial sum acceleration

X=acceleration change along X-axis between 2 data points

Y=acceleration change along Y-axis between 2 data points

Z=acceleration change along Z-axis between 2 data points

**Figure 2:**
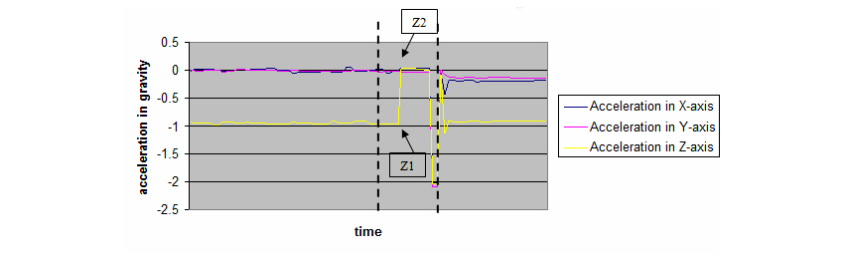
A representative graph generated from the acceleration data (the segment within the dotted line represents the period of time before and after the free fall test; Z1 is data point in time immediately prior to fall on stationary position and Z2 is the immediate data point on falling).

From the acceleration data captured during each free fall, we used Microsoft Excel software to generate the following representative graph (Figure 2).

**Figure 3:**
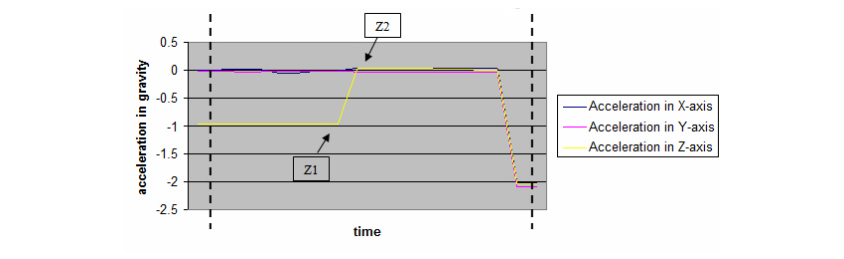
A magnified segment view of the section within the dotted lines.

Segmentation was performed to remove irrelevant noise waveforms from the representative graph in order to identify the waveform that is representative of the actual free fall. In Figure 2, we identified data point Z1 as the data point in time immediately prior to the fall in a stationary position, while Z2 is the immediate data point in time when falling. (See Figure 3 for a magnified view of the segment within the dotted line in Figure 2).

**Figure 4:**
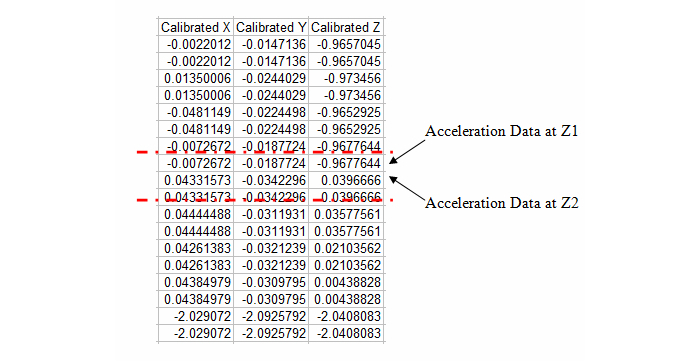
The acceleration data within the dotted line represents the duration of time prior to and after the fall captured by the triaxial accelerometer within the iPod Touch (Z1 is the acceleration data immediately before the fall and Z2 is the acceleration data at the beginning of falling).

The change in acceleration detected by the triaxial accelerometer within the iPod in the Z-axis is the absolute difference in value between data point Z1 and data point Z2. Figure 4 illustrates the acceleration data in the Excel spreadsheet within the dotted line for all 3 axes.

From Figure 4, we could then identify the corresponding acceleration data changes in the X- and Y-axis. By using the formula shown, we then determined the vectorial sum acceleration measured by Accel4Pro during the free fall. Using the same process for all the 30 trials, we ended up with the resultant mean vectorial sum acceleration, which then allowed us to proceed to perform the statistical analysis on all the acceleration data obtained.

Below is a simple illustration of how we performed the mathematical calculation to obtain the mean acceleration captured by the triaxial accelerometer housed in the iPod, using the acceleration data from Figures 2-4. Following is an example of a calculation for vectorial sum acceleration, r:

Z axis acceleration changes = 0.967764 + 0.039666 = 1.007430

Y axis acceleration changes = 0.034229 – 0.018772 = 0.015457

X axis acceleration changes = 0.043315 + 0.007267 = 0.050582

Vectorial sum acceleration, r=√(X^2^ + Y^2^ + Z^2^)

= √(0.002558 + 0.000239 + 1.014915)

= √(1.017712)

= 1.008817 g

Descriptive statistics (mean and standard deviation) were generated for the sensitivity of the device as well as comparing the acceleration data for all 3 weeks.

## Results

**Figure 5:**
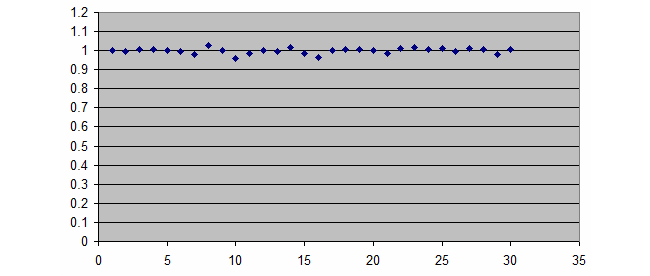
A scatter plot for all the 30 free fall trials.

The resulting descriptive statistic of mean acceleration for all the 30 trials of free fall was represented using a scatter plot as shown in Figure 5.

**Figure 6:**
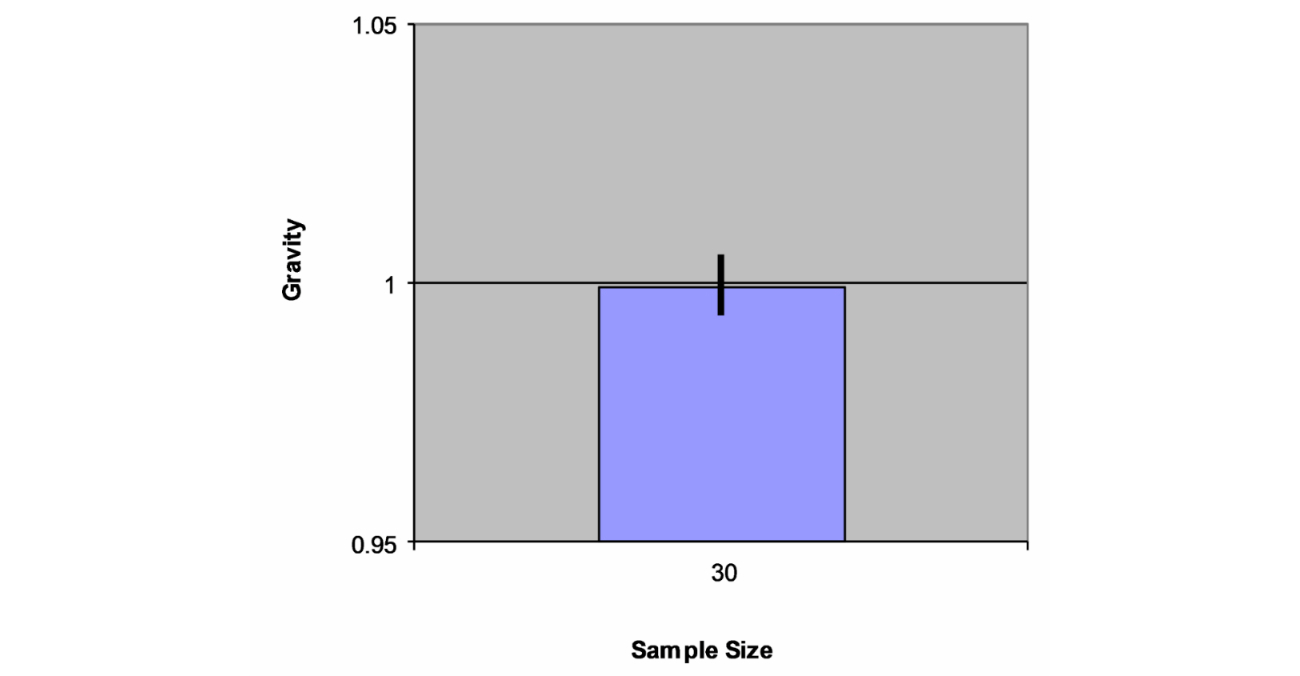
The mean vectorial sum acceleration with 95% CI 0.99-1.01.

The acceleration data captured for all 30 trials (Table 1) showed us that the mean acceleration from the trials was very accurate and consistent as evidenced by the very small standard deviation of 1.58% from a mean acceleration of 0.999 g with a 95% confidence interval of 0.993488-1.004761 (Figure 6). Week-to-week acceleration data were also found to be very consistent and accurate, indicating the reproducibility of the acceleration data captured across different times and under different conditions.

**Table 1:** Mean accelerations and standard deviations of the device as a function of the device orientation.

	1^st^ tests (G)	2^nd^ tests (G)	3^rd^ tests (G)
Trial 1	1.002297	0.984672	0.984208
Trial 2	0.995306	0.999181	1.010951
Trial 3	1.008817	0.994424	1.014362
Trial 4	1.006725	1.019200	1.007173
Trial 5	0.999943	0.983010	1.012857
Trial 6	0.995615	0.964196	0.997752
Trial 7	0.981492	1.000802	1.013392
Trial 8	1.027264	1.006944	1.005093
Trial 9	1.002452	1.008590	0.979668
Trial 10	0.960244	1.000500	1.006613
Mean (SD)	0.998016 (0.017646)	0.996152 (0.015566)	1.003207 (0.012279)
Overall mean (SD)			0.999125 (0.015094)

## Discussion

### Principal Findings

The use of mobile electronic devices in daily life has increased worldwide. Apple’s 4th generation iPod touch is one such device that has had a tremendous impact on our lives, serving as a communication device, as well as a mobile personal assistant. The built-in triaxial gyroscope and accelerometer in such devices has been shown by multiple researchers to be capable of detecting daily activities with good accuracy [1-4]. Recent literature also supports the reliability of various smartphone devices in measuring gait parameters and as an analytical tool to study balance and mobility [5-7].

Our work to determine the accuracy, sensitivity, and reproducibility of a triaxial accelerometer embedded in a 4th generation iPod Touch in relation to gravity under free fall conditions represents a pilot study for using the iPod Touch triaxial accelerometer waveforms data in a circular pattern to help clinicians recognize common gait patterns. Amick et al [8] showed recently that in a static state, the accelerometer in the iPod is capable of capturing acceleration accurately. Thus, we believe our methodology is the first of its kind in subjecting the iPod Touch to free fall testing from a height of approximately 100 cm in a controlled environment. We compared our results with the established standard of 1 g (equivalent to 9.81 m/s^2^) under these circumstances. Our results showed that the free fall acceleration data captured by the triaxial accelerometer indicated that the iPod displays a very high accuracy and sensitivity under dynamic conditions to capture acceleration data.

### Conclusions

In our opinion, the triaxial accelerometer embedded within mobile consumer communication devices has huge potential as a motion analysis tool based on its highly accurate and sensitive acceleration data output. The direction of future work should focus more on performing research involving multiple devices subjected to more complex motions over a longer duration, taking advantage of the ability to detect subtle changes in acceleration accurately. In view of its high sensitivity and accuracy under dynamic conditions, our results show that the 4th generation iPod Touch can be used in the clinical practice of joint reconstructive surgery to objectively evaluate gait changes of each patient before and after undergoing joint arthroplasty surgery.

## Abbreviations

MEMS: microelectromechanical system
